# No Evidence for Ape *Plasmodium* Infections in Humans in Gabon

**DOI:** 10.1371/journal.pone.0126933

**Published:** 2015-06-03

**Authors:** Lucresse Délicat-Loembet, Virginie Rougeron, Benjamin Ollomo, Céline Arnathau, Benjamin Roche, Eric Elguero, Nancy Diamella Moukodoum, Alain-Prince Okougha, Bertrand Mve Ondo, Larson Boundenga, Sandrine Houzé, Maxime Galan, Dieudonné Nkoghé, Eric M. Leroy, Patrick Durand, Christophe Paupy, François Renaud, Franck Prugnolle

**Affiliations:** 1 Centre International de Recherches Médicales de Franceville, CIRMF, BP 769, Franceville, Gabon; 2 MIVEGEC (UMR CNRS/IRD/UM 5290), 911 avenue Agropolis, 34394, Montpellier, Cedex 5, France; 3 CHRU de Montpellier, 39 Avenue Charles Flahault, 34295, Montpellier, France; 4 Unité de Modélisation Mathématique et Informatique des Systèmes Complexes (UMI IRD/UPMC 209), Institut de Recherche pour le Développement, 32 avenue Henr1 Varagnat, 93140, Bondy, France; 5 INRA, UMR1062 CBGP, avenue du Campus Agropolis, 34980, Montferrier-sur-Lez, France; 6 Laboratoire de Parasitologie-Mycologie, Hôpital Bichat-Claude Bernard, 46 rue Henri Huchard, 75018, Paris, France; 7 Unité Mixte de Recherche 216 IRD, Université Paris Descartes, 12 rue de l’Ecole de Médecine, 75006, Paris, France; Universidade Federal de Minas Gerais, BRAZIL

## Abstract

African great apes are naturally infected by a multitude of *Plasmodium* species most of them recently discovered, among which several are closely related to human malaria agents. However, it is still unknown whether these animals can serve as source of infections for humans living in their vicinity. To evaluate this possibility, we analysed the nature of *Plasmodium* infections from a bank of 4281 human blood samples collected in 210 villages of Gabon, Central Africa. Among them, 2255 were detected positive to *Plasmodium* using molecular methods (*Plasmodium Cytochrome b* amplification). A high throughput sequencing technology (454 GS-FLX Titanium technology, Roche) was then used to identify the *Plasmodium* species present within each positive sample. Overall, we identified with confidence only three species infecting humans in Gabon: *P*. *falciparum*, *P*. *malariae and P*. *ovale*. None of the species known to infect non-human primates in Central Africa was found. Our study shows that ape *Plasmodium* parasites of the subgenus *Laverania* do not constitute a frequent source of infection for humans. It also suggests that some strong host genetic barriers must exist to prevent the cross species transmission of ape *Plasmodium* in a context of ever increasing contacts between humans and wildlife.

## Introduction

Disease outbreaks have roughly quadrupled over the past 50 years, with 60 percent of "disease emergences" originating in animals and subsequently infecting humans. The majority of these zoonotic diseases come from wild animals, suggesting that increased fragmentation and destruction of habitats bring humans in contact with more and more pathogens, especially in biodiversity-rich regions like the tropics [1].

Because of their close genetic proximity with humans, great apes and monkeys have the potential to serve as sources of various human pathogens, as exemplified by the recent cases of AIDS and Ebola Fever [2]. The emergence of these and other diseases has been linked to the always-increasing interface between tropical wildlife communities and human populations [3]. Since most nonhuman primates live in tropical forest habitats, most interactions between humans and wild nonhuman primates occur in this high-risk interface, which has recently increased in particular because of forest encroachment [4].

The genus *Plasmodium*, responsible for malaria, one of the worst scourges of human mankind, is widespread in primates. For this and other reasons, it represents a group at risk for disease emergence in humans. First, this is a vector-borne disease and vector-borne pathogens are responsible for about one third of the new emerging infectious diseases in humans. Second, the evolutionary history of this genus has shown its potential to switch from host to host, especially between primates and humans, underlying its capacities to cross species barriers. These capacities have been highlighted in our recent history as, in 2004, another *Plasmodium* species, traditionally known to infect macaques, *P*. *knowlesi*, was reported to cause about 70% of human malaria infections in certain parts of Southeast Asia [5]. It is now considered as the “fifth human malaria parasite”. It seems now clear that this is the increasing contacts between humans and macaques that have favoured this transfer [5].

**Fig 1:**
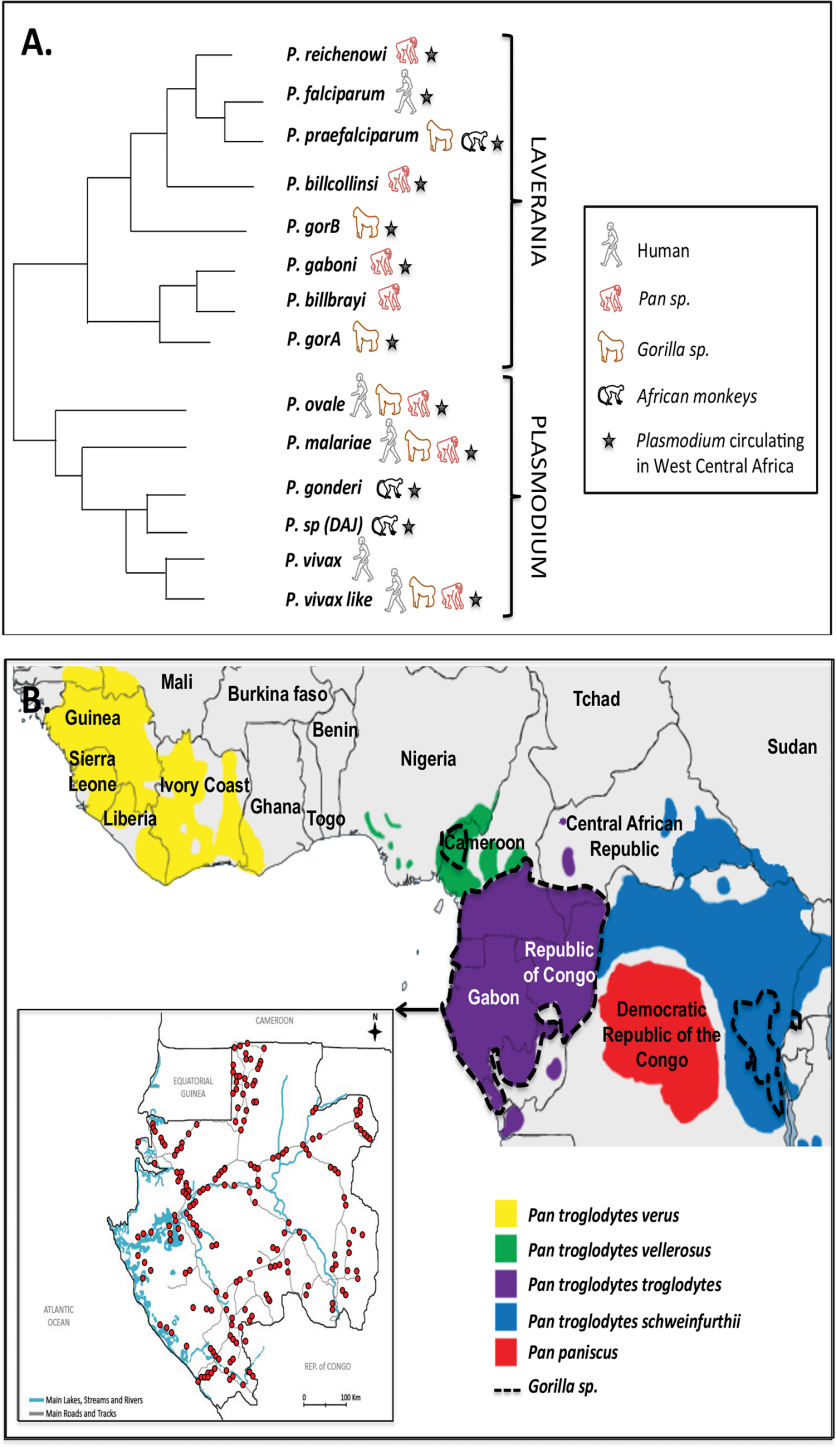
A. Schematic phylogenetic representation of relationships among *Plasmodium* species circulating in Central Africa; B. Location, in Gabon, of the 210 villages (red circles) where human blood samples were collected between 2005 and 2008. Distribution of the different sub-species of chimpanzees, bonobos and of gorillas in Central Africa is shown.

Recently, several studies have re-explored the diversity of *Plasmodium* species circulating in great apes in Africa [6–10]. They showed that gorillas and chimpanzees harboured a multitude of *Plasmodium* species, including species very closely related to the human malaria agents, *P*. *falciparum*, *P*. *malariae*, *P*. *ovale* and *P*. *vivax* (Fig 1A). Among the species related to *P*. *falciparum* and classified into the subgenus *Laverania*, three species were shown to infect only chimpanzees (*P*. *gaboni*, *P*. *billcollinsi* and *P*. *reichenowi*) and three only gorillas (*P*. *gorA*- *syn P*. *adleri*, *P*. *gorB—syn P*. *blacklocki and P*. *praefalciparum)*. But more importantly, they showed that *P*. *falciparum*, the most virulent species of all, very likely appeared following a transfer of *P*. *praefalciparum* from gorillas to humans [11]. For the species related to *P*. *malariae*, *P*. *ovale* and *P*. *vivax* (which we will refer to *P*. *malariae*-like, *P*. *ovale*-like and *P*. *vivax*-like), they are classified in the subgenus *Plasmodium*. Although for *P*. *ovale* and *P*. *malariae*, information regarding their origin are very scare if not absent, for *P*. *vivax*, recent studies suggest that its origin is either the result of a transfer to humans of parasites from Asian monkeys or African apes [9,12].

In light of these different discoveries, several authors suggested the possibility that great apes could constitute reservoirs of infections for humans in Africa [7–10]. Only one recent study, by Sundararaman et al. [13], explored this hypothesis in Cameroon and found no evidence of infections by ape *Plasmodium* in humans. Nevertheless, their work covered only a small area of the distribution of great apes in West Central Africa and in Cameroon as well. In addition, human samples concerned only a limited number of persons collected from only eight different villages.

In the present study, we evaluated the possibility of present ape-to-human transmission of *Plasmodium* species using a large bank of 4281 human blood samples collected from 210 villages of Gabon (~ 1/10 of the Gabonese villages) (Fig 1B). Gabon represents almost half of the range of western chimpanzees and gorillas in Central Africa and this country is characterized by high malaria endemicity [14]. The identification of *Plasmodium* species infecting each positive individual was performed using the next-generation sequencing 454 Titanium approach, allowing us to evaluate the zoonotic potential of ape *Plasmodium*.

## Material and Methods

### Forest-dwelling human samples

To explore the diversity of *Plasmodium* species infecting humans in Gabon, we analysed a set of 4281 blood samples (red cell clots) from an existing bank of specimens previously collected for epidemiological studies of various infectious diseases including malaria [15]. The samples were collected between June 2005 and September 2008. Over the entire period, blood collection covered 210 randomly chosen villages from the nine administrative provinces of Gabon (Estuaire, Haut-Ogooué, Moyen-Ogooué, Ngounié, Nyanga, Ogooué-Ivindo, Ogooué-Lolo, Ogooué-Maritime and Woleu-Ntem) with 10 to 34 villages per province (Fig 1B). In these 210 villages, all healthy volunteers over the age of 15 who had been residing in the village for more than one year were recruited for the study. All individuals were sampled only once.

Blood samples were usually collected in the village health care centers into 7-ml vacutener tubes containing EDTA (VWR International, France). The tubes were transported daily to the field laboratory for centrifugation (10min, 2000g). Plasma, buffy coat and red blood cells were stored separately. Samples were preserved at -20°C until the end of the field mission and then transferred on dry ice at the Centre International de Recherches Médicales de Franceville (CIRMF) and kept at -80°C until analysis. Red blood cell clots were then processed for *Plasmodium* detection.

### Ethic statement

Written consent was secured from all participants. In the case of minors, consent was obtained from at least one parent. Our study received the approval of the Gabonese Ministry of Health with a research authorization No. 00093 on these samples March 15, 2005. Note finally that all samples were analyzed anonymously.

### DNA extraction and *Plasmodium Cytb* amplification

For each sample, DNA was extracted from 200μl of red blood cell clot using the DNeasy Blood and Tissue kit (Qiagen, France) according to the manufacturer’s recommendations and eluted in 100μl of elution buffer.

To screen for *Plasmodium* infections, we tested the 4281 human samples for *Plasmodium Cytochrome b* (*Cytb*) mitochondrial sequences by a nested PCR already described in Ollomo et al. [16].

### Sample preparation for multiplex 454 sequencing

**Fig 2:**
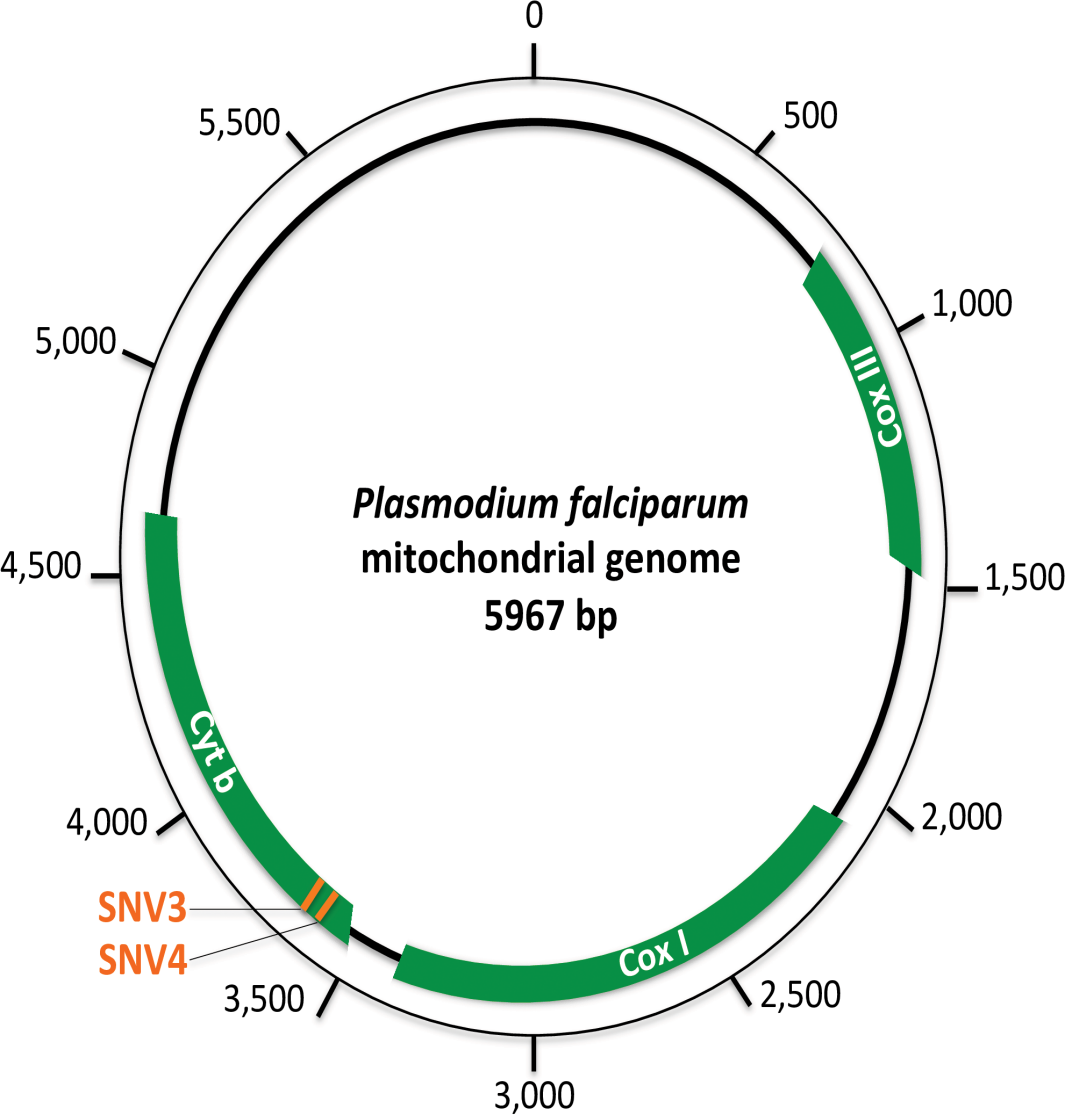
Schematic representation of the *Plasmodium falciparum* mitochondrial genome. In green are represented the three protein coding genes of the mitochondrial genome of *Plasmodium* parasites. On the *Cytochrome b* gene, used for *Plasmodium* diagnostic in this study, the position of the two SNVs (single nucleotide variations) allowing to distinguish *P*. *falciparum* from the ape *Laveranias* is shown in orange.

To identify the *Plasmodium* species present in each positive sample and detect mixed infections even when one species constitutes only a small fraction of the parasite load, we used a deep-sequencing approach. More particularly, we used the 454 GS-FLX Titanium technology (Roche) on multiplexed tagged amplicons of several hundreds of samples amplified for a fragment of 201bp of the *Cytb* gene, containing SNVs (Single Nucleotide Variants) allowing to discriminate humans from ape *Laveranias* as well as *Plasmodium* species from the subgenus *Plasmodium* (see Fig 2) [11].

The target mitochondrial fragment was amplified using the amplicons of the first round of PCR obtained during *Plasmodium* diagnostics. Based on these amplicons, a second round of amplification was performed using the following primers designed for this study (Forward: 5’-WAATTAYCCATGYCCATTRAA-3’ and Reverse: 5’-CCWGTWGCYTGCATYTATCT-3’). Forward and reverse primers were designed by adding a GS FLX Titanium Primer sequence 7bp multiplex identifiers (MID) tags published by Galan et al. [17]. Because these MID tags differed from the others by at least three substitutions, they are tolerant to several errors to avoid misassignment of reads. Every 25 μl reaction mix was composed of 12.5 μl of Multiplex PCR mix 2X (Qiagen), 2.5 μl of Q solution (Qiagen), 0.5μl of each tagged primer (10pM each) and, 1 μl of the first PCR product Amplifications were carried out in a thermal cycler using the following reaction conditions: 95°C for 15 min, followed by 35 cycles of 94°C for 30 sec, 51°C for 30 sec, 72°C for 1 min, and a final extension step of 72°C for 10 min. These tagged primers were validated for amplification of sequences of the appropriate length using *P*. *falciparum* 3D7, *P*. *malariae*, *P*. *ovale* and a set of ape *Plasmodium* (*P*. *gaboni*, *P*. *gorA*, *P*. *praefalciparum*) genomic DNA. Each isolate was amplified using a unique combination of forward and reverse MID tags. PCR amplification was confirmed visually by nucleic acid staining (EZ VISION DNA Dye, Ambresco), followed by gel electrophoresis (2% agarose in 0.5x TBE buffer) demonstrating a band of the appropriate size (~ 317bp comprising adaptors). Several positive and negative controls (no template) were used for quality assurance (see S3 Table for the list of the positive controls used in the study).

### Amplicon library preparation

The PCR products were first purified using SPRI method (solid-phase reversible immobilization) (Agencourt, AMPure XP). Then, PCR amplicon concentrations were measured using the Quant-iT PicoGreen dsDNA kit per manufacturer’s instructions (Invitrogen). Known concentrations of control DNA were prepared as directed by the Roche Technical Bulletin (454 Sequencing Technical Bulletin No. 005–2009). We assayed fluorescence intensity using a Perkin-Elmer VICTOR X3 multilabel plate reader, with fluorescein excitation wavelength of ~480nm and emission of ~520nm wavelength.

### 454 GS-FLX Titanium sequencing approach

We prepared four PCR amplicon library pools, each containing equimolar amounts of up to 700 PCR amplicons with unique MID tag combinations. These four pools were sequenced in forward and reverse directions on segregated regions of one full 454 plate using GS FLX Titanium chemistry (Roche). Sequencing was performed by Beckmann Coulter Genomics (Danvers, MA, USA).

### Sequence analysis pipeline

A custom pipeline was developed to de-multiplex, de-noise and remove PCR and sequencing artefacts from the *Cytb* reads. Sff-files obtained from each region on the 454-plate were divided into smaller isolate specific sff-files by identification of reads with exact matching MID sequences in both ends. Ambiguous primer sites were then identified (exact match) and trimmed off the flowgrams and reverse reads were reverse complemented. Forward and reverse reads were then combined to take advantage of bi-directional amplicon sequencing, since the forward read has the highest quality in the 5’-end of the target sequence, and the reverse read improves the 3’-end quality. For each individual, each read was aligned to a set of reference sequences (S1 Table) by Muscle program [18] and then BLAST against these references for species assignment using the BLASTn program. The maximal *e*-value was retained for each read. Reads were discarded if they had a similar BLAST score (similar maximal *e*-value) for two reference sequences belonging to two different *Plasmodium* species. For the others, each read was assigned to the species for which it obtained the highest score. Note that our pipeline did not allow to distinguish between the different sub-species of *P*. *ovale* (*P*. *ovale curtisi* and *P*. *ovale wallikeri*).

## Results

Of the 4281 human blood samples examined, 2255 harboured an infection with *Plasmodium* (*Plasmodium Cytochrome b* (*Cytb*) amplification). Of these, 1674 passed quality control (amplicon concentration higher than 2ng/μl) and were successfully sequenced by multiplex pyrosequencing (using the 454 GS-FLX Titanium technology, Roche). The average number of reads obtained per individual was 927.7 and 95% of them had between 285 and 1995 reads. Using our pipeline, each read was attributed to one of the reference species (S1 Table).

**Fig 3:**
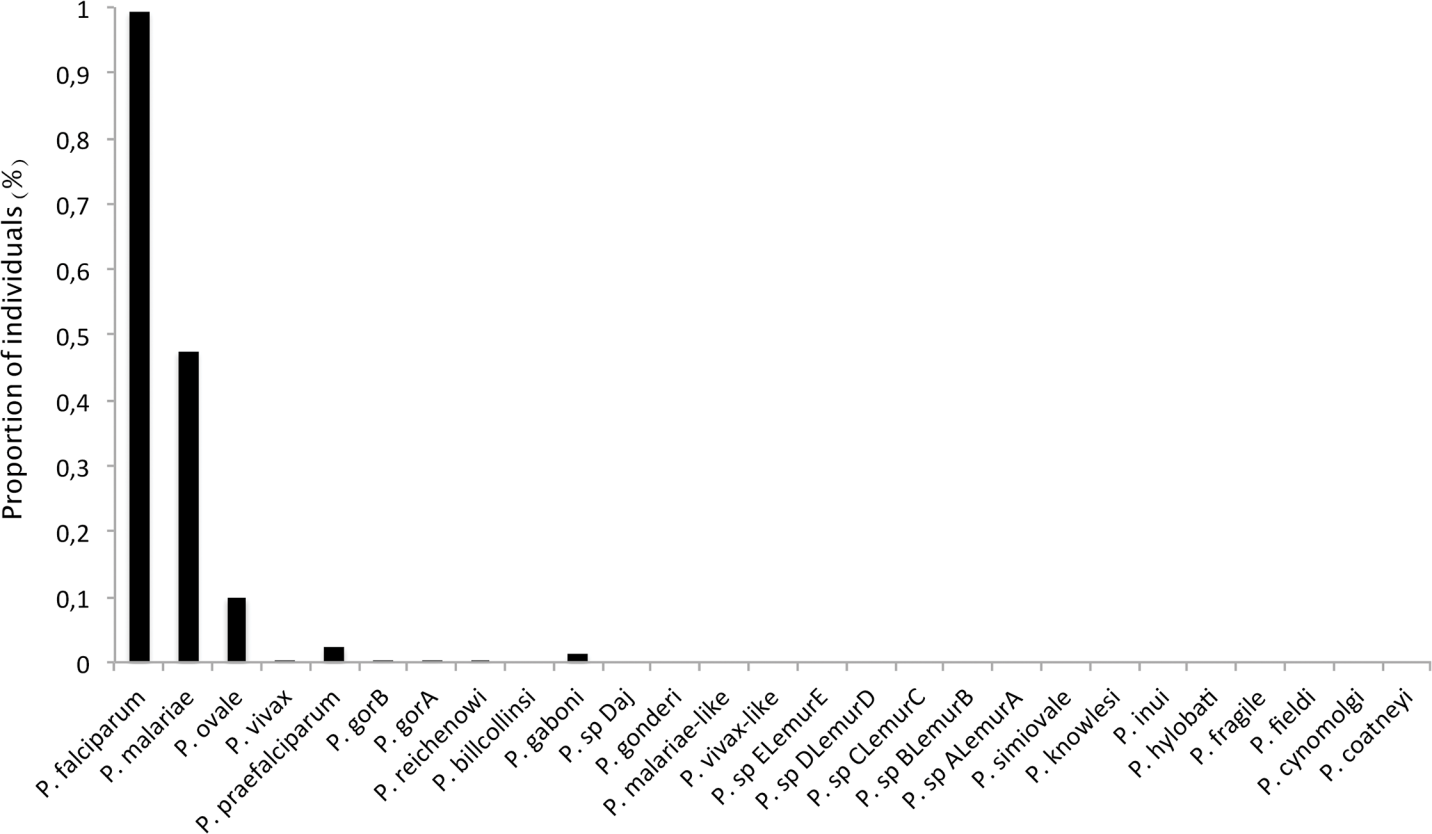
Proportion of individuals harbouring at least one read assigned to one *Plasmodium* reference species (listed in S3 Table).

The proportion of individuals harbouring at least one read assigned to one or another species is given in Fig 3. As shown, most individuals carried at least one read assigned to one of the human *Plasmodium*: 99.4% with at least one read of *P*. *falciparum*, 47.6% with *P*. *malariae* and 9.9% with *P*. *ovale*. Several individuals also carried reads belonging to *P*. *vivax*, a species supposed to be absent from Central Africa [19] as well as reads assigned to non-human primate *Plasmodium*, such as *P*. *gaboni*, *P*. *reichenowi* from chimpanzees, or *P*. *praefalciparum*, *P*. *gorA (syn*. *P*. *adleri)*, *P*. *gorB (syn*: *P*. *blacklocki*) from gorillas. Nevertheless, these species represent only 0.32% of the reads within positive individuals (range [min = 0.03%; max = 1.6%]). This thus suggests that their presence could, for their vast majority, be explained by experimental artefacts such as cross-contamination, tag switching and/or artificial mutations.

These problems become evident when observing both the negative and the positive controls. Indeed, over the four negative controls (one in each pool), one contained five “Unexpected Reads” (“URs”), belonging to *P*. *falciparum*. Regarding the eleven positive controls, which were carefully chosen because of their known single-infection, a high proportion of them (90.9%) also harboured reads belonging to a species different from the expected one (see S2 Table). This was for example the case for our *P*. *vivax* control. While this control was isolated from a patient from Thailand, several of its reads were assigned to a species only known to circulate in African apes (here *P*. *gaboni*). Similarly, in the ape-derived *Plasmodium* positive controls, reads belonging to *Plasmodium* species known to only infect the other ape species or humans were found. In these positive controls, any read belonging to another species than the one expected was therefore considered to be a “UR”. This allowed us to determine the proportions that “URs” could represent over the total number of reads obtained per individuals. On average, "URs" represented 0.5% of the reads and up to 2.5% per individuals. All other reads were assigned to the expected species.

In what follows, we explore the role these different experimental artefacts (cross-contamination, tag switching and/or artificial mutations) could play in the presence of “URs” in the Gabonese human samples.

### Cross-contamination

**Fig 4:**
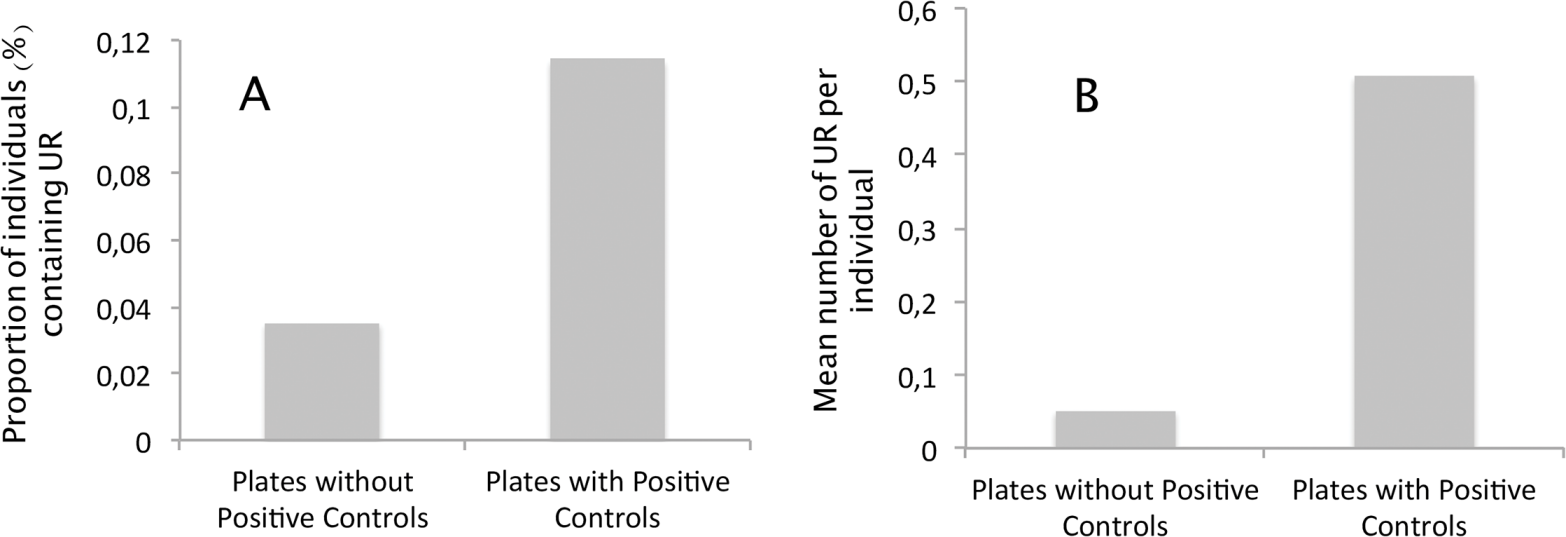
Cross-contamination estimates from the positive controls in our experimental design. A. Comparison of the proportion of individuals displaying “Unexpected Reads” (URs) (i.e reads not assigned to *P*. *falciparum*, *P*. *malariae* or *P*. *ovale*) between PCR plates where positive individuals were amplified (PCR plates with positive controls) and those without positive controls (PCR plates without positive controls). B. Comparison of the average number of “Unexpected Reads” (URs) per individual in PCR plates with (PCR plates with positive controls) and without the positive controls (PCR plates without positive controls).

One problem with nested PCRs is the possibility of cross-contaminations among wells [19]. In our experimental design, despite the precautions taken to avoid contaminations during manipulations (one-way sample flow with separated areas, preparation of DNA and PCR mix in a clean UV hood, human and ape samples treated in separate clean rooms), the presence of “URs” (*P*. *vivax* or some non-human primate *Plasmodium* species reads) in the Gabonese human samples could be due to cross-contamination from our positive controls. If this is the case, we then expect the proportion of “URs” to be higher in PCR plates where positive controls were present than in the other PCR plates (without positive controls). The second amplification round was performed for a total of 31 PCR plates. The positive controls were amplified in four of them at the end of the amplification process. We tested the prediction and, as shown in Fig 4, our results are congruent with it. Indeed, in the PCR plates containing the positive controls, we observed (i) a significantly higher proportion of human individuals containing “URs” (Logistic regression, *P-value* = 1.3 10^–6^) and (ii) a significantly higher mean number of “URs” per individual (ANOVA, *P-value =* 2.2 10^–16^).

### Tag switching

If cross-contamination can explain part of the “Unexpected Reads” (URs) in the Gabonese human samples, it cannot be at the origin of all them, especially when the DNA of the individuals never physically interacted with the positive controls (i.e. when these individuals were amplified in different PCR plates, at different times). One possibility to explain these individuals positive to “URs” could then be tag switching [20].

During amplicon pyrosequencing, we incorporated tags at both ends of the amplicons in order to recognize individuals after pooling. The sequences of the different tags and their combinations were primarily chosen to avoid such problems. Indeed, at the worst, a combination of tags was chosen to be different from another combination of tags by at least three substitutions.

After sequencing, we observed that a certain proportion of the resulting sequences possessed novel tags (unexpected tags with mutated nucleotides in the sequence). These reads were obviously discarded from the analyses since they could not be attributed to any individual. These changes in the nature of the tags can be attributable to the known high mutation rate generated by the 454 pyrosequencing process [20]. But were these artificial mutation rates high enough to generate complete switches of tags, leading to a miss-assignment of a read to an individual?

**Fig 5:**
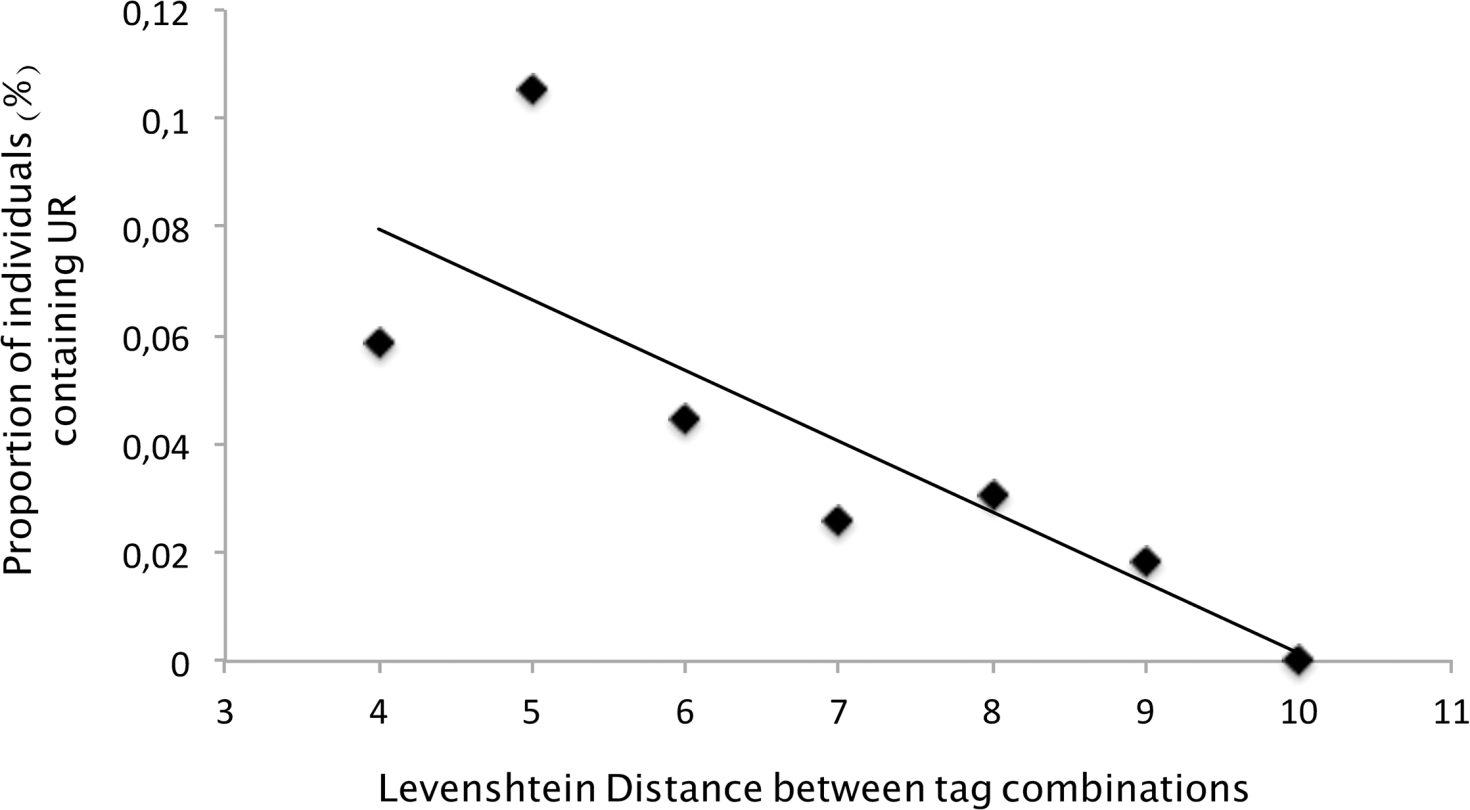
Relationship between (i) the Levenshtein distance computed between the tags of the positive controls and those of the human samples and (ii) the proportion of individuals harbouring “Unexpected Reads”.

To test this possibility, we measured the minimal genetic distance between each human sample tag combinations (using the Levenshtein Distance [21]) and the ones used for the non-human *Plasmodium* primate and *P*. *vivax* positive controls. We made the following prediction: if the artificial mutation rate was high enough to generate some switches of tags, then the proportion of tag switches from the positive controls should be higher with the human samples that display combinations of tags that were closer genetically from the positive controls. Results are given in Fig 5 and support our predictions. Indeed, in the PCR plates without the positive controls, the proportion of individuals displaying URs are significantly higher in individuals whose tags showed the smallest distance with the positive controls (Logistic regression, *P-value* = 0.001389).

### Artificial mutations

In our dataset, almost 2% of the human individuals carried at least one read assigned to *P*. *praefalciparum*. Although, to some extent, the presence of some of these reads could be explained by cross-contamination and/or tag switching from the positive controls, an ultimate phenomenon could explain such a high proportion of individuals with reads assigned to *P*. *praefalciparum*: the artificial transformation of *P*. *falciparum* sequences into *P*. *praefalciparum* reads. Indeed, given that the 454-derived mutation rate is high and that *P*. *falciparum* and *P*. *praefalciparum* only differ by two substitutions in the range of the amplified sequence, there is a possibility that artificial mutations could be at the origin of some of the *P*. *praefalciparum* reads.

**Fig 6:**
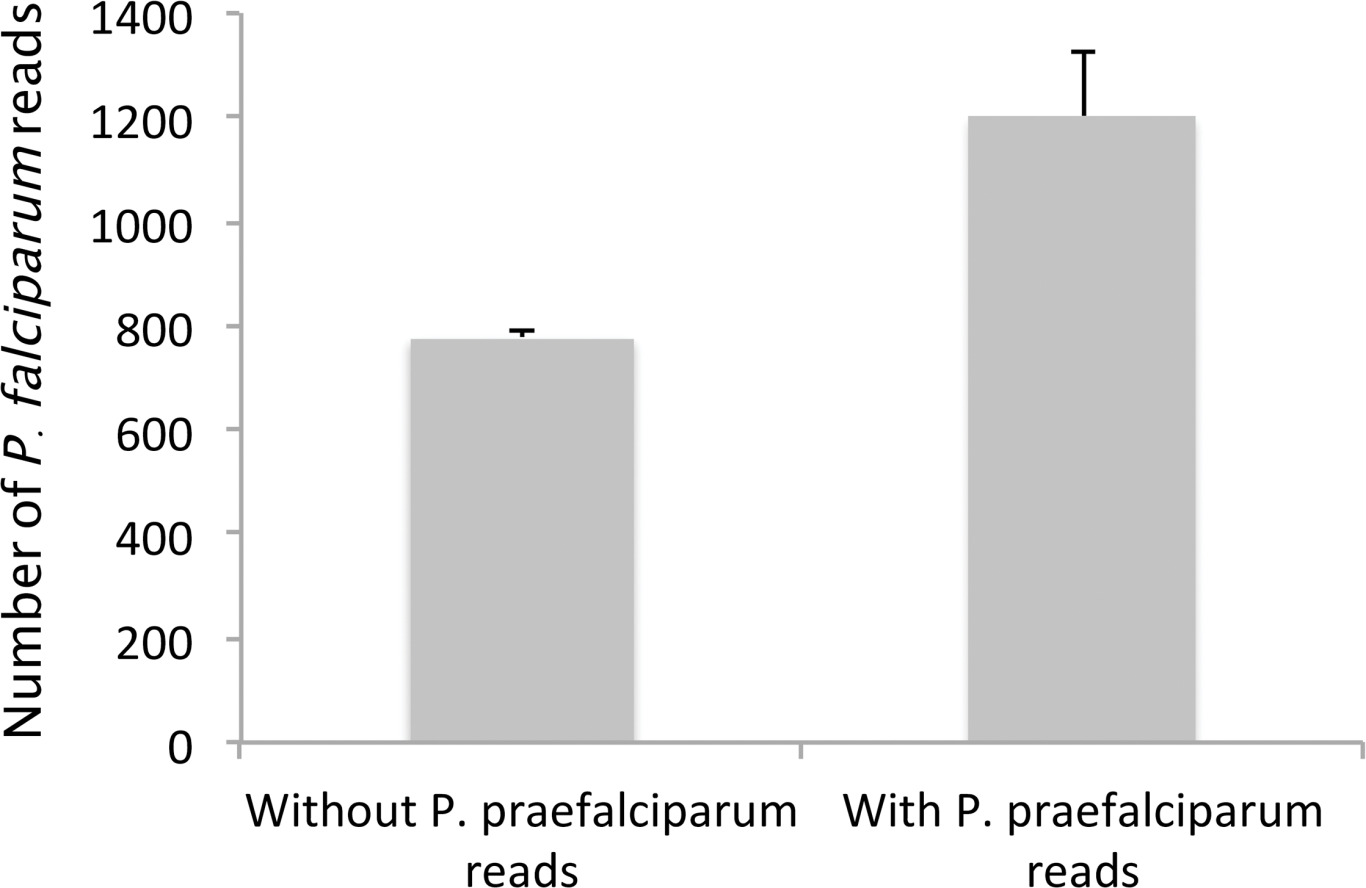
Comparison of the total number of *P*. *falciparum* reads observed in individuals displaying (i) no *P*. *praefalciparum* or (ii) more than one.

To evaluate this possibility, we analysed the relationship between the number of *P*. *falciparum* reads sequenced within individuals and the number of *P*. *praefalciparum* reads, expecting it to be positive in the case random mutation at a constant rate generate the *P*. *praefalciparum* sequences from the *P*. *falciparum* ones. Results are shown in Fig 6. They are congruent with our hypothesis as the average number of *P*. *falciparum* reads is significantly higher in individuals displaying at least one *P*. *praefalciparum* read (Fig 6, ANOVA, *P*-value = 2.16 10^–5^).

### Experimental artefacts and URs in the human samples

**Fig 7:**
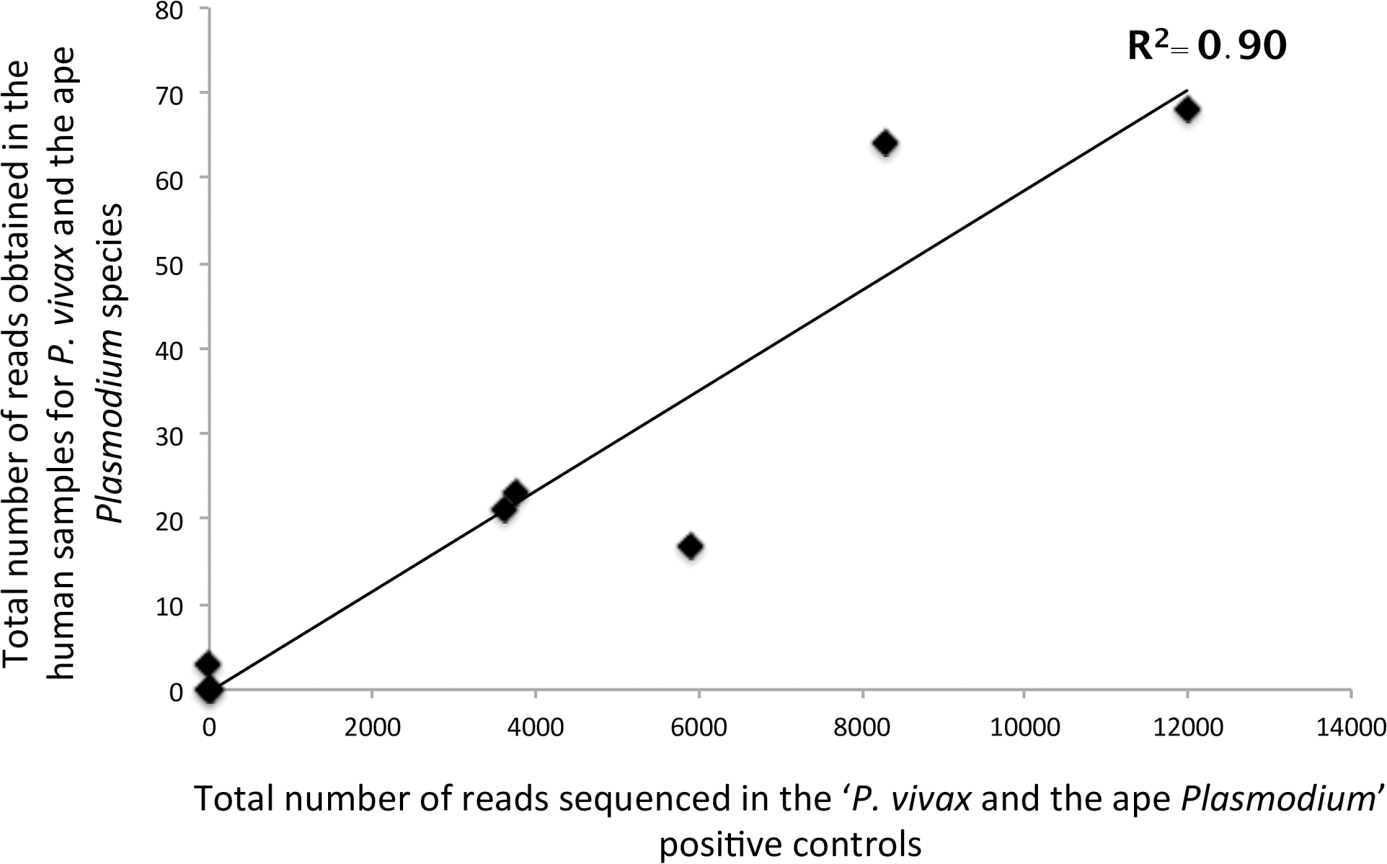
Relationship between (i) the total number of reads sequenced in the “*P*. *vivax* and ape *Plasmodium*” positive controls and (ii) the total number of reads assigned to *P*. *vivax* or an ape *Plasmodium* species in the human samples.

Finally, in order to evaluate the importance of these experimental artefacts in explaining the presence of URs in the human samples, we analysed the relationship between the total number of reads sequenced in the *P*. *vivax* and ape *Plasmodium* positive controls and the total number of “URs” (*P*. *vivax* and ape *Plasmodium*) obtained in the human samples. As shown in Fig 7, the number of reads in the positive control is strongly significantly positively correlated with the total number of URs observed in humans, explaining almost 90% of the variations (Spearman correlation test, *P-value* = 0.0046, *R* ^*2*^ = 0.90).

### Human infections

Removing low-frequency reads within each individual is one proposed solution (e.g. [20]) to counteract the different biases generated by this kind of experimental protocol. Based on the percentages of unexpected reads (“URs”) detected within our positive controls (up to 2.5%), we here took a conservative 5% as cut-off value. This cut-off value represents the lowest proportion of reads one species must have within an individual to be considered as present in this individual.

Using this cut-off, Gabonese individuals are only infected with *P*. *falciparum*, *P*. *malariae* and *P*. *ovale*. We estimated the prevalence of *P*. *falciparum*, *P*. *malariae*, *P*. *ovale* and of the co-infections within our sample. Over the 1674 positive samples, 76.6% contained only *P*. *falciparum*, 6.3% *P*. *malariae* and 0.71% *P*. *ovale*. Mixed infections represented 16.4% of the samples among which 14.9% were *P*. *falciparum / P*. *malariae*, 0.53% *P*. *falciparum / P*. *ovale*, 0.24% *P*. *malariae* / *P*. *ovale* and 0.72% *P*. *falciparum / P*. *malariae / P*. *ovale*. None of the samples contained *P*. *vivax* or ape *Plasmodium*.

## Discussion

### No evidence of non-human primate *Plasmodium* infecting humans in Gabon

Over 4281 samples distributed all over Gabon, a country representing a large part of the range of western chimpanzees and gorillas, we explored the diversity of *Plasmodium* species infecting human populations living in a forested environment. Of the 1674 *Plasmodium* positive blood samples examined using Next Generation Sequencing methods, there was no evidence of zoonotic infections with any of the six *Laverania* species known to circulate in apes (Fig 1A). Only three *Plasmodium* species historically recognised as human *Plasmodium* were detected in our sampling: *P*. *falciparum*, *P*. *malariae* and *P*. *ovale*.

Our results are congruent with results of a recent study realised in Cameroon on a far smaller sampling, in which no evidence of ape *Laverania* infections was detected as well [13]. Our study, in addition to the previous one, suggests therefore that the transmission of non-human primate *Plasmodium* to humans is very rare in Central Africa (if not absent), even in populations living in close contact with apes.

Why are such transfers rare? One explanation could be that the vectors of non-human primate *Plasmodium* are not anthropophilic and cannot therefore serve as bridge between humans and non-human primates. This seems however to be at odds with several recent observations. Indeed, although only little information is available on the potential vectors of ape *Plasmodium* [22], among the sylvan mosquito species that were, up to date, found to be infected with ape *Plasmodium*, one species was *Anopheles moucheti*. This species is known to be anthropophilic, a major human *Plasmodium* vector in Central Africa and could thus easily play the role of bridge vector between apes and humans [22]. In addition, at least one transfer of ape *Plasmodium* to human was formerly documented in Africa (a case of an ape *P*. *vivax*) [9], thus highlighting the possibility that some mosquito species indeed may play the role of bridge between the two categories of hosts.

One explanation could thus be a strong host specificity preventing the transmission of parasites from one host to another. Several experiments of artificial transplantations were made in the past with human and ape *Laverania* species and always failed to induce either an infection or malaria symptoms even in splenectomised animals [10]. Invasion of red cells by the parasites is governed by a set of surface protein interacting with proteins expressed at the surface of the host cells [23]. Several studies suggest that this interaction could be very specific and explain the strong host tropism. In particular, several authors proposed that the interaction between the *Plasmodium* protein EBA 175 and the host-specific forms of Glycophorin A (GPA) could be responsible in *Laverania* parasites of the strong host specificity [24]. However, a recent study questions the implication of the EBA175-GPA proteins as sole responsible for the host tropism and proposes that species-specific differences in the interaction between *Plasmodium* RH5 and host Basigin could also be involved. Another recent study suggests that other *plasmodium* RH genes could also be involved in host specificity or adaptation to the vertebrate host [25].

Beyond *Laverania* species apes (gorillas and chimpanzees) are also naturally infected with species of the subgenus *Plasmodium*, namely *P*. *malariae*, *P*. *ovale* and *P*. *vivax*-like parasites [8,9,11,26,27]. Species of this subgenus are known to be naturally more prone to infect a multiplicity of host species. Thus, several of these species in particular those infecting apes (*P*. *malariae-like*, *P*. *ovale-like and P*. *vivax-like*) have been shown to be able to induce symptomatic malaria in humans in experimental or accidental infections [9,28]. Our results do not show any evidence of ape to human *P*. *vivax* transfers in Gabon. For ape *P*. *malariae* and *P*. *ovale*, no study has examined their molecular characteristics compared to their human equivalent which make it impossible so far to distinguish them from the human ones. Complementary studies should therefore be done to be able to estimate their zoonotic potential.

### No evidence of human *P*. *vivax* circulation in Gabon

Our results finally suggest the absence of human *P*. *vivax* naturally circulating in human populations in Central Africa. This is in agreement with previous studies that also failed to molecularly detect *P*. *vivax* from Central African populations despite immunological evidences of exposure [13,19]. In addition, it has to be noticed that the only transfer of *P*. *vivax-like* from apes to humans has been described in a European traveller in Central African Republic [9]. In this context, this absence of human *P*. *vivax* naturally circulating in Central Africa is very likely the result of the high prevalence of Duffy negativity observed in the Central African populations, which is known to confer a high level of resistance to humans against this parasite.

Nevertheless, there is a risk that the situation does not remain the same forever. As previously discussed, great apes constitute a reservoir of *P*. *vivax* in Central Africa and transfers to humans have been documented in certain occasions [9]. In addition, the contacts between humans and primates are constantly increasing due to logging, forest encroachment and illegal conservation of animals as pets and there is also more and more evidence that the parasite could evolve alternative pathways to infect Duffy negative persons [29–31]. Finally, non-African Duffy positive human populations live in these regions of the world (immigrants, workers) and are fully susceptible to *P*. *vivax*.

## Supporting Information

S1 Table Reference sequences used to determine the origin of the 454 reads. (DOCX)

S2 Table Number of reads obtained per *Plasmodium* species for the different positive and negative controls in the four pools. (XLSX)

S3 Table Positive controls used in the study. (DOCX)
