# Supplementary material for: No Evidence for Ape Plasmodium Infections in Humans in Gabon
Source: PLoS One. 2015 Jun 3;10(6):e0126933. doi: 10.1371/journal.pone.0126933 (PMC4454650; doi:10.1371/journal.pone.0126933)
Supplement: S1 Table — (DOCX) [file pone.0126933.s001.docx]

**Table S1. Reference sequences used to determine the origin of the 454 reads.**

| **Reference name** | **Species** | **Accession number** |
| --- | --- | --- |
| PbillcollinsiHM235351 | *P.billcollinsi* | HM235351 |
| PbillcollinsiHM235360 | *P.billcollinsi* | HM235360 |
| PbillcollinsiHM235390 | *P.billcollinsi* | HM235390 |
| PbillcollinsiHM235392 | *P.billcollinsi* | HM235392 |
| PbillcollinsiHM235395 | *P.billcollinsi* | HM235395 |
| PbillcollinsiGQ355477 | *P.billcollinsi* | GQ355477 |
| PbillcollinsiGQ355478 | *P.billcollinsi* | GQ355478 |
| PbillcollinsiGQ355479 | *P.billcollinsi* | GQ355479 |
| PspchimpanzeeC3HM234976 | *P.billcollinsi* | HM234976 |
| PbillcollinsiHM235325 | *P.billcollinsi* | HM235325 |
| PcoatneyiAB354575 | *P.coatneyi* | AB354575 |
| PcynomolgiAY800108 | *P.cynomolgi* | AY800108 |
| PfalciparumACBS01001974 | *P.falciparum* | ACBS01001974 |
| PfalciparumAJ298775 | *P.falciparum* | AJ298775 |
| PfalciparumAY282924 | *P.falciparum* | AY282924 |
| PfalciparumAY282947 | *P.falciparum* | AY282947 |
| PfalciparumAY282957 | *P.falciparum* | AY282957 |
| PfalciparumGQ355472 | *P.falciparum* | GQ355472 |
| PfalciparumGQ355473 | *P.falciparum* | GQ355473 |
| PfalciparumGQ355474 | *P.falciparum* | GQ355474 |
| PfalciparumKC175310 | *P.falciparum* | KC175310 |
| PfalciparumKC175311 | *P.falciparum* | KC175311 |
| PfalciparumKC175312 | *P.falciparum* | KC175312 |
| PfalciparumKC175313 | *P.falciparum* | KC175313 |
| PfalciparumKC175314 | *P.falciparum* | KC175314 |
| PfalciparumKC175315 | *P.falciparum* | KC175315 |
| PfalciparumKC175316 | *P.falciparum* | KC175316 |
| Pfalciparum3D7AY282930 | *P.falciparum* | AY282930 |
| PfieldiAB354574 | *P.fieldi* | PfieldiAB354574 |
| PfieldiAB444132 | *P.fieldi* | PfieldiAB444132 |
| PfieldiAB444133 | *P.fieldi* | PfieldiAB444133 |
| PfragileAB444136 | *P.fragile* | PfragileAB444136 |
| PgaboniHM235279 | *P.gaboni* | HM235279 |
| PgaboniHM235309 | *P.gaboni* | HM235309 |
| PgaboniHM235332 | *P.gaboni* | HM235332 |
| PgaboniHM235335 | *P.gaboni* | HM235335 |
| PgaboniHM235337 | *P.gaboni* | HM235337 |
| PgaboniHM235346 | *P.gaboni* | HM235346 |
| PgaboniHM235348 | *P.gaboni* | HM235348 |
| PgaboniHM235349 | *P.gaboni* | HM235349 |
| PgaboniHM235353 | *P.gaboni* | HM235353 |
| PgaboniHM235363 | *P.gaboni* | HM235363 |
| PgaboniHM235398 | *P.gaboni* | HM235398 |
| PgaboniHM235404 | *P.gaboni* | HM235404 |
| PgaboniFJ895307 | *P.gaboni* | FJ895307 |
| PgaboniGQ355468 | *P.gaboni* | GQ355468 |
| PgaboniGQ355470 | *P.gaboni* | GQ355470 |
| PgonderiAB434918 | *P.gonderi* | AB434918 |
| PadleriHM235278 | *P.gorA* | HM235278 |
| PadleriHM235281 | *P.gorA* | HM235281 |
| PadleriHM235297 | *P.gorA* | HM235297 |
| PadleriHM235313 | *P.gorA* | HM235313 |
| PadleriHM235372 | *P.gorA* | HM235372 |
| PblacklockiHM235294 | *P.gorB* | HM235294 |
| PblacklockiHM235304 | *P.gorB* | HM235304 |
| PhylobatiAB354573 | *P.hylobati* | AB354573 |
| PinuiGQ355482 | *P.inui* | GQ355482 |
| PinuiGQ355483 | *P.inui* | GQ355483 |
| PinuiAB354572 | *P.inui* | AB354572 |
| PinuiAB444109 | *P.inui* | AB444109 |
| PknowlesiAY722797 | *P.knowlesi* | AY722797 |
| PmalariaeAB489194 | *P.malariae* | AB489194 |
| PmalariaeAB354570 | *P.malariae* | AB354570 |
| PmalariaeDRCJGQ355486 | *P.malariae* | DRCJGQ355486 |
| Pmalariae-PttHM235345 | *P.malariae-like* | HM235345 |
| PspPttKC175322 | *P.malariae-like* | KC175322 |
| PovalecurtisiHQ712052 | *P.ovale* | HQ712052 |
| PovalecurtisiAB354571 | *P.ovale* | AB354571 |
| PovalewallikeriHQ712053 | *P.ovale* | HQ712053 |
| PovalewallikeriKC175307 | *P.ovale* | KC175307 |
| MO454JF923762 | *P.praefalciparum* | F923762 |
| MOEBJF923761 | *P.praefalciparum* | JF923761 |
| PpraefalciHM235273 | *P.praefalciparum* | HM235273 |
| PpraefalciHM235276 | *P.praefalciparum* | HM235276 |
| PpraefalciHM235292 | *P.praefalciparum* | HM235292 |
| PpraefalciHM235306 | *P.praefalciparum* | HM235306 |
| PpraefalciG1HM235274 | *P.praefalciparum* | G1HM235274 |
| PpraefalciG1HM235288 | *P.praefalciparum* | G1HM235288 |
| PreichenowiAJ251941 | *P.reichenowi* | AJ251941 |
| PreichenowiHM235328 | *P.reichenowi* | HM235328 |
| PreichenowiHM235394 | *P.reichenowi* | HM235394 |
| PsimiovaleAB434920 | *P.simiovale* | AB434920 |
| PspALemurHQ712054 | *P.sP.Alemur* | HQ712054 |
| PspBLemurHQ712055 | *P.sP.BLemur* | HQ712055 |
| PspCLemurHQ712056 | *P.sP.CLemur* | HQ712056 |
| PspDAJ-2004MandrillAY800112 | *P.sP.Daj* | AY800112 |
| PspDLemurHQ712057 | *P.sP.DLemur* | HQ712057 |
| PspELemurJN131536 | *P.sP.ELemur* | JN131536 |
| PvivaxAY598060 | *P.vivax* | AY598060 |
| PvivaxAY598119 | *P.vivax* | AY598119 |
| PvivaxY17721 | *P.vivax* | Y17721 |
| PvivaxAmouchetiAJX444726 | *P.vivax-like* | JX444726 |
| PvivaxAmouchetiBJX444724 | *P.vivax-like* | JX444724 |
| PvivaxAmouchetiCJX444725 | *P.vivax-like* | JX444725 |
| PvivaxBprimeJX444723 | *P.vivax-like* | JX444723 |
| PvivaxBumbiJX444722 | *P.vivax-like* | JX444722 |
| PvivaxColaJX444719 | *P.vivax-like* | JX444719 |
| PvivaxEuropeantravelerJX444721 | *P.vivax-like* | JX444721 |
| PvivaxKarlaJX444720 | *P.vivax-like* | JX444720 |
| PvivaxSal-1NC007243 | *P.vivax-like* | NC007243 |
| Pvivax-likechimpanzeeGQ355481 | *P.vivax-like* | GQ355481 |
| Pvivax-likeGggHM235311 | *P.vivax-like* | HM235311 |
| Pvivax-likeGggKC175317 | *P.vivax-like* | KC175317 |
