# Supplementary material for: No Evidence for Ape Plasmodium Infections in Humans in Gabon
Source: PLoS One. 2015 Jun 3;10(6):e0126933. doi: 10.1371/journal.pone.0126933 (PMC4454650; doi:10.1371/journal.pone.0126933)
Supplement: S3 Table — (DOCX) [file pone.0126933.s003.docx]

**Table S3. Positive controls used in the study.**

| **Positive control name** | **Geographic origin** | **Host species** | ***Plasmodium* species** |
| --- | --- | --- | --- |
| B' | Gabon | Chimpanzee | *P. gaboni* |
| Ebène | Gabon | Chimpanzee | *P. gaboni* |
| GG04 | Gabon | Gorilla | *P. gorA* |
| MO454 | Gabon | *Cercopithecus nictitans* | *P. praefalciparum* |
| MOEB | Gabon | Gorilla | *P. praefalciparum* |
| olkMcpM | Gabon | Chimpanzee | *P. gaboni* |
| SL69 | Sierra Leone | Chimpanzee | *P. reichenowi* |
| Pfalci | French guiana | Human | *P. falciparum* |
| Pmalariae | Ivory Coast | Human | *P. malariae* |
| Povale | Central African Republic | Human | *P.ovale* |
| Pvivax | Soudan | Human | *P. vivax* |
